# Identification of *Porrocaecum moraveci* in red kites in England and Wales, a species of conservation concern

**DOI:** 10.1007/s00436-025-08512-1

**Published:** 2025-06-10

**Authors:** William S. Funk, Tammy Shadbolt, Mark T. Fox, Anthony W. Sainsbury, Damer P. Blake

**Affiliations:** 1 Royal Veterinary College, 4 Royal College St, London, NW1 0TU UK; 2 Zoological Society of London, Outer Circle, Regent’s Park, London, NW1 4RY UK; 3 Pathobiology and Population Science, Royal Veterinary College, 4 Royal College St, London, NW1 0TU UK; 4 Pathobiology and Population Science, Royal Veterinary College, Hawkshead Lane, North Mymms, AL9 7TA UK; 5 WildCare Oklahoma, 8505 Wildwood Ln, Noble, OK 73068 USA

**Keywords:** Gastrointestinal parasites, *Milvus milvus*, Nematodes, *Porrocaecum*, Red kites, Translocation

## Abstract

The population of free-living red kites (*Milvus milvus* [Linnaeus 1758]) in England and Wales has increased since 1989 as a consequence of species reintroduction. The red kite, however, remains of conservation concern, with populations in Europe considered to be in decline. Plans to translocate birds from England to Spain have been initiated, prompting consideration of the disease risks associated with the translocation of parasites which may be present within the source population. This study utilized published morphological markers and molecular polymerase chain reaction techniques to identify archived adult helminth parasites extracted from the gastrointestinal tract of red kites found dead and examined post-mortem in England and Wales between 2014 and 2021. Helminths of the genus *Porrocaecum* (Railliet and Harry 1912) were identified in 22 out of the 23 helminth-infected red kites from a wide geographical distribution, suggesting that this parasite is common in the red kite population in England and Wales. Molecular characterization using internal transcribed spacer 2 (ITS-2) and 28S rDNA sequences identified *Porrocaecum moraveci* (Gu et al. 2023), the first report of this recently described species in the UK. *Ascaridia* (Dujardi 1845) sp., *Capillaria* (Zeder 1800) sp., and *Syngamus trachea* (Montagu 1811) ova were also detected during the post-mortem examinations (PMEs) and are known to be present within European red kite populations, suggesting that these parasites do not represent a novel disease risk to the destination population in Spain. Previous reports of *Porrocaecum angusticolle* (Molin, 1860) in British and other European red kite populations should now be revisited to confirm identity and assess the risk of parasite translocation.

## Introduction

Red kites (*Milvus milvus* [Linneaus, 1758]) are medium-sized, free-living raptors (Accipitridae) that are widely distributed throughout much of Europe, residing in farmlands, mixed and broadleaf forests, and edges of urban areas (Cross and Davies 2005). Red kites were heavily persecuted and almost extirpated from Great Britain in the early 1900s. A collaborative reintroduction program was initiated by the Royal Society for the Protection of Birds (RSPB) and English Nature (now Natural England (NE)) to translocate birds from Spain and Sweden to England (Evans et al. 1999). Since the initiation of the program in 1989, the population in England has increased to over 3000 breeding pairs, representing more than 10% of the world’s red kite population (Molenaar et al. 2017). In contrast, populations in Spain, Portugal, Germany, and France are considered to be in decline (Birdlife International 2020). In 2022, plans to translocate free-living red kite chicks from England to Spain commenced through a multi-partner collaboration between NE, RSPB, the Zoological Society of London (ZSL), the Roy Dennis Foundation, the Forestry Commission, Acción por el Mundo Salvaje (AMUS), and authorities in Andalucía and Extremadura (Common et al. 2024).

The Disease Risk Analysis and Health Surveillance (DRAHS), a partnership project between NE and ZSL, carries out post-mortem examinations (PMEs) of submitted free-living red kites found dead in England and Wales as part of the ongoing post-release health surveillance for the species. The findings of these PMEs are essential for informing disease risk analysis (DRA) for the newly initiated translocation of birds from England to Spain. During translocation, there is the potential for previously unknown, potentially novel, parasites present within the source population to be inadvertently introduced to the final destination and cause disease within a population of naïve animals (Hartley and Sainsbury 2017). An understanding of which parasites may be present or absent within the two populations, source and destination, is therefore important in estimating the disease risks associated with these translocations.

Gastrointestinal helminths are frequently detected during post-mortem examination of red kites submitted to the DRAHS program. However, the identification of the species of helminths has not been confirmed in these birds, except for a single published case study of a red kite, considered to have died of electrocution, in which a heavy infection of adult *Porrocaecum angusticolle* (Molin, 1860) nematodes (identified based on morphology) was detected in the large intestine and was associated with partial obstruction (Peniche et al. 2011). Within mainland Europe, other nematode species have been reported in red kites including *Capillaria tenuissima* (Rudolphi 1809), *Cyrnea mansion* (Seurat, 1914), *Eucoleus dispar* (Dujardin, 1845, López-Neyra, 1947), *Microtetrameres* (Travassos 1915) sp., *Physaloptera alata* (Rudolphi 1819), *Porrocaecum depressum* (Zeder 1800), *Procyrnea leptoptera* (Rudolphi 1819, Chabaud 1958), *Synhimantus affinis* (Seurat 1916), *S. lateceps* (Rudolphi 1819), and *Trichinella pseudospiralis* (Garkavi 1972) (Ileesacs-Gomez et al. 1993; Sanmartin et al. 2004; Honisch and Krone 2008; Blanco et al. 2017; Marucci et al. 2021). No trematode species have been identified in free-living red kites in England and Wales, but *Neodiplostomum* (Ralliet 1919) sp., *Metorchis* (Looss 1899) sp., and *Strigea* (Abildgaard 1790) sp. have been identified in red kites in Germany (Krone 2000). Similarly, no cestode species have been identified nor reported in free-living red kites in England and Wales. Blanco et al. (2017) found unidentified cestode ova in the feces of red kites collected at supplemental feeding sites in Spain but did not confirm active infection and noted that only nematodes were detected in the feces of kites that fed almost exclusively on wild prey.

Here, we present, to the best of our knowledge, the first descriptive study to identify helminths present within the free-living red kite population in England and Wales using morphological and molecular methods. We report the use of morphological markers and molecular polymerase chain reaction (PCR) techniques to identify selected helminths present within the gastrointestinal (GI) tract of free-living red kites found dead and submitted to the DRAHS project for PME between 2014 and 2021. We report on the distribution of identified helminth species within England and Wales and note whether the detected helminth species are known to exist within mainland European populations with the goal of identifying parasites which could pose a novel disease risk to destination populations as a consequence of translocation.

## Study area

Samples for this study were collected between 2014 and 2021 from England and Wales. The climate of England and Wales is temperate with a high variability in weather throughout the regions, with the southern and eastern regions being generally drier, warmer, and less windy (Meteorological Office n.d.). Mean annual temperatures range between 8.0 and 10.0 °C in the Midlands to 10.5–12.0 °C in the Southwest. Annual precipitation ranges between less than 550 mm in the southeast to more than 3200 mm in the northwest with consistent precipitation events year-round. Geography ranges greatly throughout the region. The topography of England and Wales ranges from coastal lowlands at elevations of 0 m above sea level in parts of England to 1085 m in the mountainous upland areas of Wales. Land use in England and Wales is predominantly agricultural, forestry, open land, urban, or water.

## Methods

### Case selection

Records of free-living red kites found dead in England and Wales and submitted to the DRAHS project for PME between 2014 and 2021 were reviewed. Upon submission to the project, each kite was assigned a unique identifier. Red kites assessed for this study were selected based on the gross detection of adult helminth parasites within the GI tract. PMEs conducted during the study period followed a standardized avian PME protocol (Molenaar 2008). In brief, as part of the PME, the entire GI tract was dissected and grossly inspected, and the blunt side of a scalpel blade was used to scrape the mucosa. Adult helminths identified in the GI tract were collected as pooled samples in sterile polypropylene universal tubes (Starlab, Milton Keynes, UK), preserved in 70% ethanol (EtOH) (Kimia, Witham, UK), labeled with the carcass reference number, and archived at room temperature. Following PME, red kite carcasses were retained and frozen at − 20 °C. For the purpose of this study, tubes containing adult helminths for all case birds were retrieved from the archive and subjected to further examination.

### Morphological characterization

Prior to identification, the pooled samples were divided into individual tubes (one helminth per tube) and given specific identification numbers. Individual samples were placed in baths of commercial lactoglycerol (Microscience, Newcastle upon Tyne, UK) until they were sufficiently clear for identification. The time required for adequate clearing varied between samples and ranged from 1 min to over an hour. After clearing, the samples were observed using a Leica EZ40HD stereomicroscope and photographed at 2048 × 1536 pixels resolution. Measurements were taken using Fiji ImageJ using a standardized scale at each magnification level* (*141 pixels/millimeter (p/mm) at 8 ×, 187p/mm at 10 ×, 235p/mm at 12.5 ×, 308p/mm at 16 ×, 388p/mm at 20 ×, 460p/mm at 25 ×, 556p/mm at 30 ×, and 646p/mm at 35 ×). Measurements taken included body length, maximum body width, esophagus length, cecum length (when applicable), spicule length, and tail length. Ratios between body and esophagus lengths and esophagus and cecum lengths were also calculated. Morphological identification to the genus level was undertaken using published keys (Anderson et al. 1974).

### Molecular characterization

Thirty-seven nematodes were selected for DNA extraction following morphological characterization, ensuring that at least one sample from each bird was included. Nine of these nematodes were physically intact to permit morphological identification and measurements to be taken (six identified as *Porrocaecum* sp. (five females, one male) and three identified as *Ascaridia* sp. (one female, two males)). The additional 28 samples were utilized to permit identification of otherwise unidentifiable samples. Extraction was performed using a Qiagen DNeasy Blood and Tissue DNA kit according to manufacturer’s instructions (Qiagen, Manchester, UK). Initially, primers targeting the nematode internal transcribed spacer 2 (ITS-2) sequence were used for pan-nematode molecular identification, using Nemat_ITS2_NC1 (5′-ACGTCTGGTTCAGGGTTGTT-3′) and Nemat_ITS2_NC2 (5′-TTAGTTTCTTTTCCTCCGCT-3′) (Gasser et al. 1993). Primers were synthesized by Merck (Haverhill, UK). PCR was performed according to manufacturer’s protocols for MyTaq DNA Polymerase. Specifically, 12.5 µL of 2 × MyTaq mix (Bioline, London, UK; polymerase and buffer), 0.1 µL of each primer at 100 µM, 11.3 µL of molecular grade water (Invitrogen, Paisley, UK), and 1 µL of template DNA were combined to create a 25 µL total reaction volume. Cycling conditions for the ITS-2 assays consisted of denaturation at 95 °C for 1 min, then 94 °C/30 s, 54 °C/30 s, 72 °C/1 min for 35 cycles, and a final extension at 72 °C for 7 min. A segment of the mitochondrial cytochrome c oxidase subunit I (COI) was amplified using the primers Nemat_COI_LCO (5′-GGTCAACAAATCATAAAGATATTGG-3′) and Nemat_COI_HCO (5′-TAAACTTCAGGGTGACCAAAAAATCA-3′) to generate new sequence resources (Folmer et al. 1994). Cycling conditions for the COI assay consisted of denaturation at 95 °C for 1 min, then 94 °C/30 s, 50 °C/30 s, 72 °C/1 min for 35 cycles, and a final extension at 72 °C for 7 min. After initial sequence analysis, a third assay was undertaken targeting a segment of the 28S rDNA using a subset of seven samples with primers 28S-F (5′-AGCGGAGGAAAAGAAACTAA-3′) and 28S-R (5′-ATCCGTGTTTCAAGACGGG-3′) to validate species identification (Nadler et al., 1998). Cycling conditions were the same as for the COI assay, with the exception that the annealing temperature was increased to 56 °C. Electrophoresis was performed utilizing 50 mL of 1.5% ultrapure agarose gel and 1 × Tris–Borate-EDTA (TBE) buffer (both Invitrogen). Six microliters of Safe View nucleic acid stain (NBS Biologicals, Huntingdon, UK) were added to each gel. Following electrophoresis at 50v, the PCR products were visualized using a Syngene U-Genius Imaging System (Syngene, Cambridge, UK).

Following confirmation of amplification, amplicons were purified using a Qiagen PCR purification kit following the manufacturer’s protocols and sent to Eurofins or Source Bioscience for Sanger sequencing (Eurofins, Konstanz, Germany; Source Bioscience, Nottingham, UK). Sanger sequence assembly, curation, and analysis were performed using CLC Main Workbench v8.1 with default parameters. Following curation, sequences were identified by comparison to those available in public repositories using the Basic Local Alignment Search Tool (BLAST) from the National Library of Medicine, National Center for Biotechnology Information (NLM, NCBI). Standard nucleotide BLASTn was conducted using default parameters. Reference sequences identified by BLASTn were downloaded and aligned with new sequences using CLC Main Workbench with the “very accurate option” default parameters. Alignments were curated manually, trimming and editing gaps by comparison to the consensus for each sequence, and exported in fasta format for phylogenetic analysis using MEGA X (Kumar et al. 2018), including maximum likelihood (ML, optimal model identified using the minimum Bayesian information criterion in MEGA X), neighbor joining (NJ), and unweighted pair group method with arithmetic mean (UPGMA) methods, with 1000 bootstrap iterations. Alignments including paired ITS-2 and 28S sequences per species were concatenated using CLC Main Workbench and analyzed as above. All sequences are available from GenBank under the accession numbers OR359357-OR359369 (ITS-2), OR356064-OR356077 (COI), and PQ634575-PQ634581 (28S).

### Statistics and spatial visualization

Statistical analysis of the parasite infections was conducted using R × 64 version 4.1.3 with standard software packages. Fisher’s exact test was used to identify any association between age and sex of the red kites and the presence of grossly detected parasites.

ArcGIS Pro version 3.3.0 was utilized for spatial visualization of the geographical distribution of helminth species identified in red kites admitted to the study. Point data from the National Grid Reference coordinates relating to the location where each bird was collected from was utilized to map out the distribution of red kites with and without adult parasitic infections.

## Results

### Case analysis

**Fig. 1:**
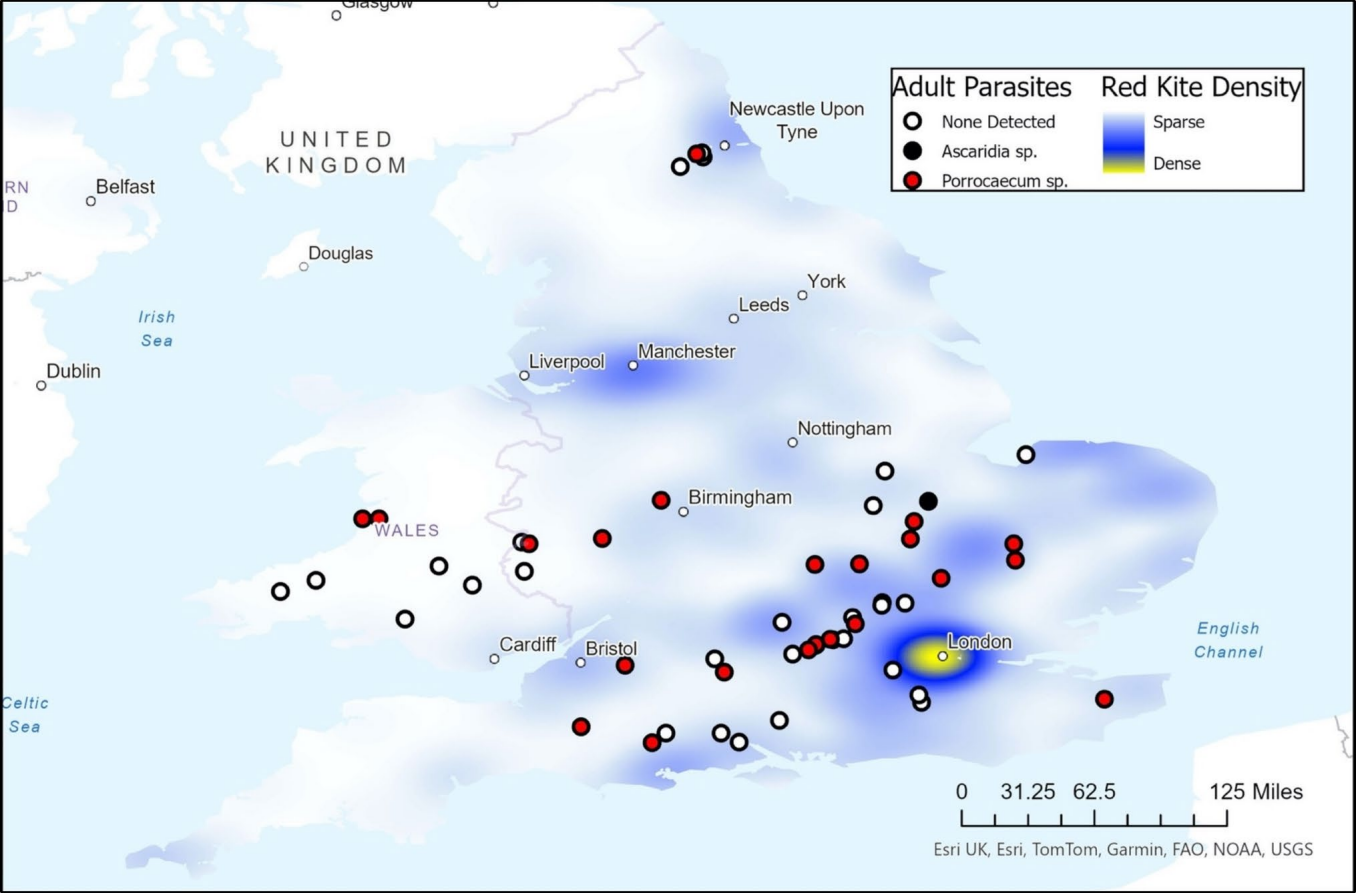
Map showing the locations of red kites submitted to the disease risk analysis and health surveillance (DRAHS) project between 2014 and 2021 illustrating where identified adult nematode parasites were detected (*n* = 23). Red kite density calculated utilizing location data of red kites submitted to Ebird (a citizen science project through Cornell University) up to May 2024 (Ebird 2024), visualized using the heat map function in ArcGIS Pro 3.3.0 with dynamic methodology at a radius of 20

Fifty-three free-living red kites found dead were submitted to the DRAHS project between 2014 and 2021 and subjected to PME (Fig. 1); parasitology results are reported in Tables 1 and 2. Twenty-three of these red kites were recorded as hosting adult helminths within the GI tract on gross pathological examination and were therefore selected as cases for this study; all parasites were grossly identified as nematodes. There was no significant relation between age (*p* = 0.225) or sex (*p* = 0.328) and the presence of grossly detectable nematodes. A total of 165 nematodes were recovered from these 23 kites at PME and archived. The mean number of nematodes recorded within each kite was 5.77 (intensity of infection; *n* = 22) and range 1 to 28 nematodes (*n* = 22). One kite reported to contain more than 100 nematodes within the GI tract was excluded from this analysis since the exact number was not stated in the post-mortem report. Post-mortem examination reports pertaining to these 23 birds did not identify the species of adult nematodes present, although it was noted that microscopic examination of GI matter had been conducted and nematode ova were detected in 11 of the 23 birds. Reports based on ova detection identified nine kites found to be infected with *Capillaria* sp., one case found to be infected with both *Capillaria* sp. and *Syngamus* trachea, and one case found to be infected with *Ascaridia* sp. No reports identified cestode or trematode ova in any of the assessed cases.

**Table 1:** Age, gender, body condition, and degree of autolysis of red kites assessed by the DRAHS project between 2014 and 2021 and associated cases of parasitic infection (*n* = 53). *HY* hatch year, *AHY* after hatch year

	Age Group				Gender			Body Condition					Degree of Autolysis			
	Undetermined (*n* = 1)	Juvenile (*n* = 7)	HY Adult (*n* = 14)	AHY adult (*n* = 31)	Undetermined (*n* = 16)	Male (*n* = 17)	Female (*n* = 20)	Undetermined (*n* = 2)	Poor (*n* = 9)	Moderate (*n* = 13)	Good (*n* = 25)	Very good (n = 4)	Fresh (*n* = 10)	Mild (*n* = 12)	Moderate (*n* = 5)	Severe (*n* = 26)
Parasites detected	1	2	8	12	6	7	10	0	6	6	8	3	4	5	3	11
No Parasites detected	0	5	6	19	10	10	10	2	3	7	17	1	6	7	2	15

**Table 2:** Summary of red kites submitted to the disease risk analysis and health surveillance (DRAHS) program and their respective parasite burden (# = number) as reported by the findings of this study and post-mortem examination (PME) reports (excluding the single individual from 2021 with an unspecified number of adult worms in excess of 100 in the PME report)

Year	# of kites submitted	# of kites infected with adult nematodes	Proportion of kites infected with adult nematodes	# of kites with helminth ova	Range of adult parasite burden in infected kites	Mean parasite burden in infected kites
2014	3	2	0.66	1	3–9	6
2017	2	0	0	1	0	0
2018	21	6	0.28	0	2–28	8.33
2019	13	8	0.62	2	3–13	9.75
2020	1	0	0	1	0	0
2021	13	7	0.53	6	1–7	3.57

### Morphological characterization of adult GI nematodes

**Fig. 2:**
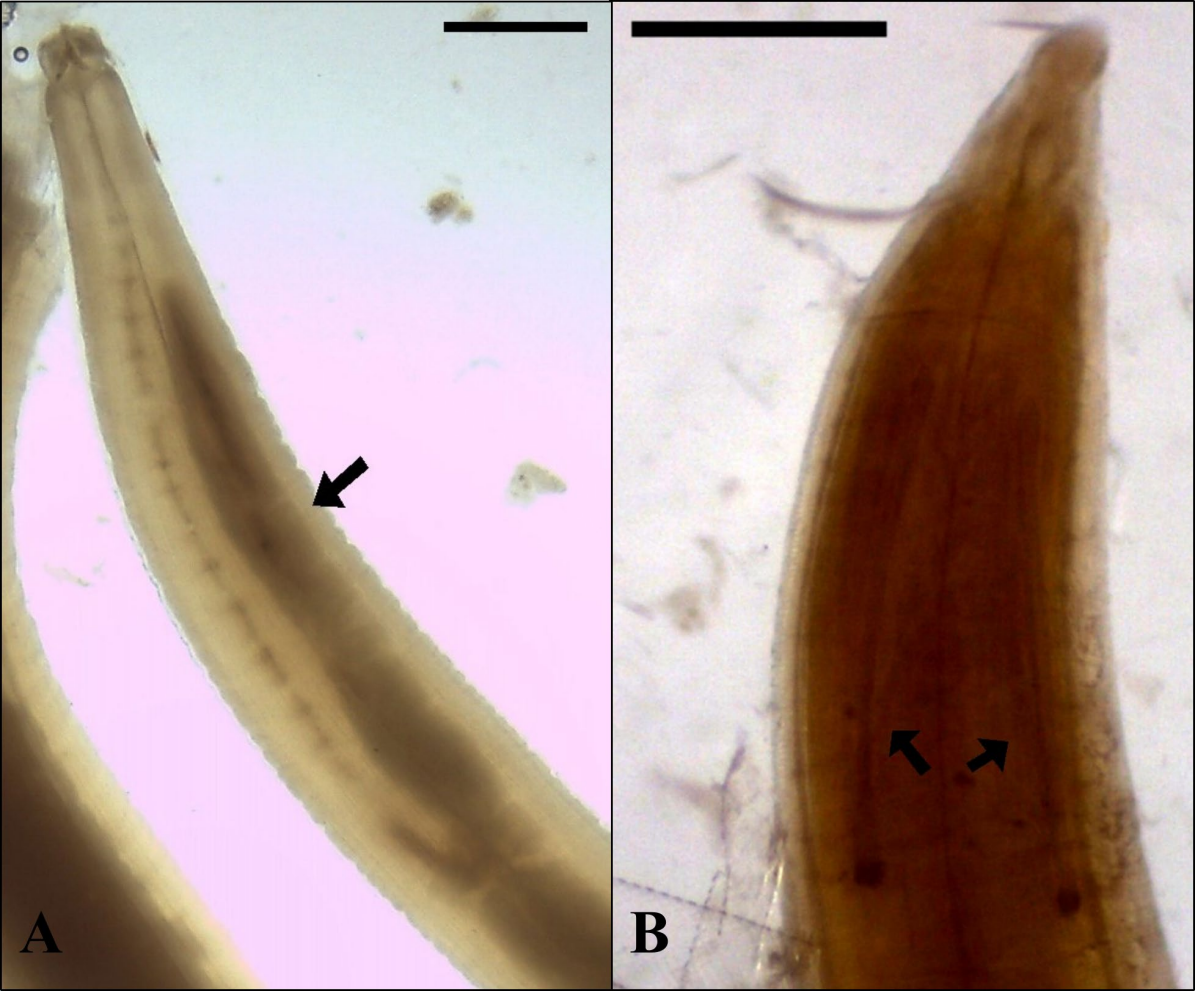
**A** *Porrocaecum moraveci* anterior end (8 ×) with a clearly defined intestinal cecum (arrow). Scale bar is equal to 1 mm. **B** *Porrocaecum moraveci* posterior end (35 ×) with unevenly sized spicules (right spicule measures 1.00 mm, 1.83% of body length; left spicule measured 1.02 mm, 1.87% of body length). Scale bar is equal to 0.5 mm. Color adjusted to allow for better visualization of spicules

Ninety-eight of the 165 adult nematodes retrieved from the archives and examined using light microscopy had morphological characters intact that permitted visual identification to genus level. Eighty-seven nematodes were suitable for various metrics of morphometric analysis, with the full range of measurements collected from 46 nematodes. Twenty-two of the 23 kites were found to be infected with the nematodes of the genus *Porrocaecum* (*n* = 95 nematodes) (Fig. 2) and just one red kite was infected with nematodes of the genus *Ascaridia* (*n* = 3 nematodes). None of the kites were infected with trematode or cestode species.

The nematodes identified as *Porrocaecum* sp. were large nematodes with transversely striated cuticle, off-white to light yellow in color. They had a maximum width around the middle of the body, a long intestinal cecum (being almost two-thirds the length of the esophagus), a conical tail in both sexes, and spicules of unequal lengths noted on some (but not all) samples. Morphometrics are listed in Table 3. The nematodes identified as *Ascaridia* sp. were large nematodes with transversely striated cuticle, light pink in color. They had a maximum width around the middle of the body, a long esophagus with no cecum, and a conical tail in both sexes; morphometric analysis presented as range (mean) with all measurements in mm. Two males were identified with a body length of 51.70–54.70 (53.03), maximum width of 0.91–1.17 (1.04), esophagus length of 1.77–2.15 (1.96) representing 9.51–12.52% (11.02) of the body length, spicule length measuring 0.91–1.34 (1.12), and the tail measuring 0.33–0.34 (0.34). A single female was identified with a body length of 77.17, maximum width of 1.82, esophagus length of 11.79 representing 15.27% of the body length, and a tail measuring 0.38.

**Table 3:** Comparative morphometrics of *Porrocaecum angusticolle* and *P. moraveci* (in mm). *BL* length of body, *EL* length of esophagus, *EB* ratio of esophagus to body length, *CL* cecum length, *CE* ratio of cecum to esophagus, *SL* spicule length, *TL* tail length

Characteristics	Current study (*P. moraveci*)		Gu et al. 2023 (*P. moraveci*)		Guo et al. 2021 (*P. angusticolle*)		Sanjota et al. 2013 (*P. angusticolle*)		Barus et al. 1978 (*P. angusticolle*)		Mazgovoi 1953 (*P. angusticolle*)		Morgan & Schiller 1950 (*P. angusticolle*)	
	Male (*n* = 16)	Female (*n* = 27)	Male (*n* = 1)	Female (*n* = 2)	Male (*n* = 9)	Female (*n* = 6)	Male (*n* = 10)	Female (*n* = 8)	Male (*n* = n/a)	Female (*n* = n/a)	Male (*n* = n/a)	Female (*n* = n/a)	Male (*n* = n/a)	Female (*n* = n/a)
BL	27.78–98.58	34.76–124.20	62	117.0–138.0	42.0–105	65.0–127.0	11.0–26.0	22.0–33.0	40–144	75.0–196.0	62.0–87.0	83.0–128.0	34.0–78.0	38.0–140.0
EL	1.56–12.52	1.45–11.79	3.61	4.68–6.61	1.97–5.65	2.65–5.30	3.1–3.36	3.0–3.77	2.7–5.72	4.14–6.43	2.80–3.74	4.32–5.97	3.80–6.10	4.00–6.40
EB (%)	3.22–23.03	1.85 −16.49	5.8	4.00–4.80	3.79–6.62	4.00–4.80	12.9–28.2	11.4–13.6	3.97–6.75	3.28–5.52	4.30–4.52	4.66–5.20	7.82–11.2	4.60–10.5
CL	0.87–3.50	1.10 −5.64	2.66	2.85–4.52	1.45–4.50	1.80–3.71	1.5–1.6	1.6–1.8	1.55–4.5	2.9–5.4	–	2.97–3.89	2.40–3.30	2.60–3.80
CE (%)	49.39–79.87	45.08–82.55	73.7	60.9–68.5	69.7–83.8	65.7–71.4	47.6–48.4	47.7–53.3	57.4–78.7	70.1–84.0	–	65.16–68.75	54.1–63.2	59.4–65.0
SL	0.27–1.47	–	0.70	–	0.51–1.18	–	1.7–1.9	–	0.575–1.21	–	0.68–0.91	–	0.90–1.30	–
TL	0.25–0.84	0.30 −1.19	0.33	0.50–0.60	0.24–0.35	0.46–0.69	–	0.33–0.57	0.2–0.4	0.41–0.94	0.30–0.38	0.41–0.69	0.24–0.30	0.26–0.40

### Molecular characterization of GI nematodes

**Fig. 3:**
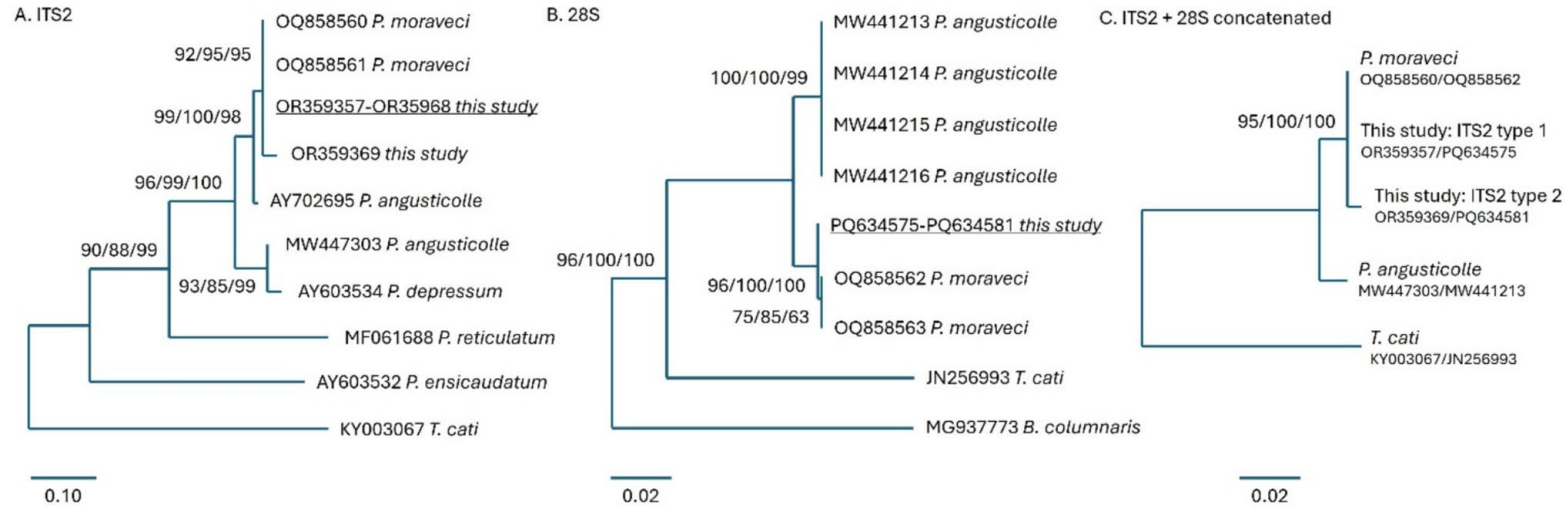
Optimal *m*aximum *l*ikelihood (ML) phylogenetic comparison of *Porrocaecum* species*:* **A** internal transcribed spacer 2 (ITS-2), **B** 28S rDNA, and **C** concatenated ITS-2 + 28S rDNA sequences with references available from GenBank. **A** For ITS-2, 21 sequences were included representing an alignment of 544 bp, analyzed using the Kimura 2-parameter model with gamma distribution to calculate evolutionary distance. **B** For 28S rDNA, 15 partial 28S sequences were included representing an alignment of 746 bp, analyzed using the Kimura 2-parameter model with invariant sites. **C** Concatenated sequences analyzed using the Kimura 2-parameter with uniform rates. Each alignment was trimmed to permit comparison with published reference sequences. GenBank accession numbers are shown, with sequences indicated with an “OR” or “PQ” prefix generated in this study. Support for each node is presented, indicating outcomes from ML/neighbor-joining/unweighted pair group method with arithmetic mean methods when more than 80% of replicate trees presented the same relationship in at least one measure

Microscopic identification of adult nematodes to the genus level was confirmed, and the *Porrocaecum* species identified by PCR and amplicon sequencing; PCR was attempted on all three samples of *Ascaridia* sp., but no amplicons were produced. Amplicons were produced from 13 of the 37 *Porrocaecum* sp. nematodes tested using the ITS-2 assay (313 bp) and 14 using the COI assay (626 bp), including 8 nematodes with positive reactions for both. The parasite genomic DNA used as a template exhibited varied levels of degradation between individuals, likely due to delays between the time of kite death and sample preservation, limiting PCR amplification from many. All amplicons were sequenced and used for parasite identification by comparison using BLASTn with the GenBank non-redundant nucleotide database. Two ITS-2 sequence types of the anticipated length were detected, including 12 nematodes represented by sequence type 1 (OR359357-68) and 1 nematode with sequence type 2 (OR359369). Both sequence types were most closely matched with *P. moraveci*, a *Porrocaecum* species recently described from the Eurasian marsh harrier (*Circus aeruginosus* [Linnaeus 1758]; accession number OQ858560-1; sequence identities type 1: 100%, 100% coverage, type 2: 97%, 100% coverage). The close relationship with *P. moraveci* was confirmed by phylogenetic comparison with other *Porrocaecum* species sequences (maximum likelihood, Kimura 2-parameter with gamma distribution, Fig. 3A).

Four COI sequence types were detected, including 2 to 7 nematodes per sequence type. No equivalent *Porrocaecum* sequences were available in GenBank for comparison as the primers used had not previously been applied to the genus. Instead, all COI sequence types were most closely matched with *Baylisascaris columnaris* (Leidy 1851; sequence identities 91.53–91.85%, accession number KY580736.1), although sequence similarity was not high. Two *Porrocaecum* species were represented by COI sequences in GenBank prior to this study (*P. angusticolle*: MW440991.1, MW440990.1, MW440989.1, MW 440988.1, and *P. reticulatum* [Linstow 1899]: MF113244.1), but neither showed significant similarity with the sequences produced in this study since they represent a different segment of the COI gene than that sequenced here, providing new COI sequence resources for *Porrocaecum* species (Guo et al. 2021). In response, PCR was attempted using primers targeting the same COI amplicon described in the latter paper, with no amplicons produced (not shown).

For additional validation, a fragment of the 28S rDNA was amplified from seven isolates, yielding a single sequence type (759 bp, PQ634575-81) that was most closely comparable to *P. moraveci* (OQ 858562–3, 99.87% identity, 100% coverage). Phylogenetic comparison confirmed this relationship, highlighting distinct sequence variation from *P. angusticolle* (maximum likelihood, Kimura 2-parameter with invariant sites, Fig. 3B). ITS-2 (types 1 and 2) and 28S sequences from this study were concatenated and compared to reference sequences to improve confidence, reinforcing identification as *P. moraveci* (maximum likelihood, Kimura 2-parameter with uniform sites, Fig. 3C).

## Discussion

This study reports for the first time, to the best of our knowledge, the results of morphological and molecular identification of adult helminths extracted from the GI tract of a large cohort of free-living red kites. Our results suggest that *P. moraveci* infection is common within the red kite population in England and Wales, as 22 of the 23 red kites infected with helminths were infected with *Porrocaecum* species parasites, of which those successfully identified were all *P. moraveci*. The only previous reports of red kites have described *P. angusticolle*, a close relative of the recently described species *P. moraveci* that is common in many raptor species (Illesacs-Gomez et al. 1993; Sanmartin et al. 2004; Richardson and Kinsella 2010; Santoro et al. 2010; Peniche et al. 2011; Guo et al. 2021; Gu et al. 2023). While *P. angusticolle* has not been detected in this study, its absence from the UK cannot be confirmed given the small scale of the sample set examined.

*Porrocaecum moraveci* is a recently described species that bears many physical similarities to *P. angusticolle* (Gu et al. 2023). The type specimens were collected from the intestinal tract of a Western marsh-harrier in the Czech Republic as part of a helminth survey. This paper is the second to identify *P. moraveci* based on morphology and molecular analysis, further supporting the classification of *P. moraveci* as a separate species. ITS2 sequencing revealed two closely related sequence types that formed a single well-defined phylogenetic clade with sequences published for *P. moraveci*, although only one was identical. Sequencing from the 28S locus confirmed annotation of all successfully sequenced nematodes as *P. moraveci*. Recognition of this new species recommends re-examination of other red kite populations for *Porrocaecum* infection, as re-annotation might be required. For example, the GenBank record AY702695 attributed to *P. angusticolle* demonstrated an intermediate genotype more closely associated with *P. moraveci* than other *P. angusticolle* ITS-2 sequences (Fig. 3A). It should be noted that this sample does not have an associated publication, so it is uncertain whether morphological identification was utilized on this sample, suggesting that reannotation might be required given the more recent description of *P. moraveci*. Though the case numbers were limited, visualization of the red kite submission locations showed that the distribution of red kites in which adult *P. moraveci* was identified (*n* = 22) matched well with the distribution of all red kites submitted to DRAHS for PME between 2014 and 2021 (*n* = 53, Fig. 1), suggesting that *P. moraveci* is uniformly distributed throughout the bird’s range in England and Wales. The limited sample size available to this study precluded statistical analysis of parasite occurrence over time and location, and the limited number of successful PCR isolates may have masked the identification of other closely related species.

This study detected an infection of mature *Ascaridia* sp. in a single case. Analysis of post-mortem examination reports suggested that an additional kite had been identified at PME as excreting *Ascaridia* species ova, but no mature nematodes were identified. Analysis of PME reports also suggested that nine kites had been found to be shedding *Capillaria* sp. ova at PME and one kite shedding both *Capillaria* sp. and *Syngamus trachea* ova; however, mature nematodes were not identified at the time of examination. Both *Capillaria* sp. and *Syngamus trachea* have been identified in raptors within Spain; however, not specifically in red kites (Illescas-Gomez et al. 1993; Sanchez-Andrade et al. 2002; Sanmartin et al. 2004). As *Ascaridia* sp., *Capillaria* sp., and *Syngamus trachea* were only identified through this study in a limited number of birds, it is not possible to conclude, with confidence, whether these parasite species are prevalent throughout the range of the red kite in England and Wales or whether their prevalence is changing over time.

There have been approximately 40 species of *Porrocaecum* species described in the literature, although the lifecycle and genetic relationships of these species are poorly understood (Guo et al. 2021; Gu et al. 2023). *Porrocaecum angusticolle* has been identified in the GI tract of birds belonging to the Accipitriform and Strigiform families in North America, Europe, and Asia (Guo et al. 2021; McAllister et al. 2022; Liu et al. 2023). The recent description of *P. moraveci* from a Eurasian marsh harrier and now red kites suggests past descriptions of *P. angusticolle* should be revisited (Gu et al. 2023). While the lifecycle of *P. moraveci* has not been described in the literature, other closely related species such as *P. angusticolle* have been identified in intermediate hosts such as earthworms and insectivores (Moravec 1971; Portoles et al. 2004). The majority of studies investigating *P. angusticolle* have focused on morphology and molecular characterization, not whether infection was associated with disease. However, it has been noted that *Porrocaecum* species can burrow into the mucosa of the gastric wall, forming hemorrhagic lesions, ulcerations, and hematomas, and severe infections may stunt growth and result in death of the host (Liu, 2023).

As noted above, several studies have used morphometrics to identify *P. angusticolle* in free-living, wild avian species (Baylis and Daubney 1922; Yamaguti 1935; Yamaguti 1941; Morgan and Schiller 1950; Mazgovoi 1953; Hartwich 1959; Hartwich 1979; Sanjota et al. 2013; Guo et al. 2021) including red kites (Barus et al. 1978). Morphometric analyses varied between studies and included nematode body length, esophagus length, dorsal lip length and width, subventral lip length and width, cecum length, spicule length, caudal papillae arrangement, egg size, distance from vulva opening to anterior end, and tail length. A subset of these morphometrics has been included in Table 3. Overall, the morphometrics presented in the study are within the same range as those listed in this table, highlighting the similarities between *P. angusticolle* and *P. moraveci*. However, Sanjota et al. (2013) report notably shorter body length and larger spicules compared to other publications. It should also be noted that all referenced publications have relatively low sample sizes, and some do not list the sample size at all. There is a high level of variation in the measurements between the reported ranges, which many attributed to a several variables, including individual variation, sample desiccation, host autolysis, equipment sensitivity, measurement methods, sample size, and methods of preservation and clearing. This is an unfortunate artifact of utilizing samples collected from free-ranging wildlife.

Multiple studies have reported *P. angusticolle* in raptors in Spain, including red kites (Illescas-Gomez et al. 1993; Sanchez-Andrade et al. 2002; Sanmartin et al. 2004). Illescas-Gomez et al. (1993) identified *P. angusticolle* in two red kites and one black kite (*Milvus migrans* (Boddaert 1783)) from 125 raptors of various species that had died at a rehabilitation center in the southeast of Spain between 1985 to 1988. Since only two red kites were examined, the likely distribution of *P. angusticolle* across the population is unclear. Additionally, they appear to have used strictly morphological identification methods. As several *Porrocaecum* species are morphologically similar, this identification may be inaccurate with some or possibly all really being *P. moraveci* (Guo et al. 2021; Gu et al. 2023). Sanmartin et al. (2004) conducted a similar study on 285 raptors representing 14 species, sourced from four rehabilitation centers in Galicia between 1991 and 1996. These authors identified several parasite species, though not *P. angusticolle*, in the single red kite case included in their study. However, they did identify *P. angusticolle* in common buzzard (*Buteo buteo* [Linnaeus 1758])*,* European sparrowhawk *(Accipiter nisus* [Linnaeus 1758])*,* Northern goshawk *(Accipiter gentilis* [Linnaeus 1758])*,* European kestrel (*Falco tinnunculus* [Linnaeus 1758)])*,* barn owl *(Tyto alba* [Scopoli 1769]), tawny owl (*Strix aluco* [Linnaeus 1758])*,* black kite*,* and European honey buzzard (*Pernis apivorus* [Linnaeus 1758])*.* Sanchez-Andrade et al. (2002) conducted fecal analysis for parasitic ova from 142 raptors in rehabilitation centers in Galicia, including a single red kite. The ova found in the feces of the red kite were unidentified, but authors reported finding *Porrocaecum* species ova in 14.8% of the samples examined from other species.

While these studies are limited in confirming enzootic infection of *Porrocaecum* species in Spanish red kites, it is the opinion of the authors that there is enough evidence to assume that *Porrocaecum* sp. is an enzootic parasite in compatriot Spanish raptors. The studies examined above have identified *P. angusticolle* in Spanish raptors for over 35 years, in both the northwest and the southeast of Spain. Genetic analysis of *Porrocaecum* sampled in England and Wales identified *P. moraveci*, suggesting colonization by a different species. However, in the absence of genetic confirmation of species identity of the Spanish *Porrocaecum* population, it cannot be ruled out that *P. moraveci* is present. The only genetic sequences of *P. angusticolle* in GenBank are from populations in the Czech Republic (MW447303.1, MW447304.1, MW447305.1, MW441213.1, MW441214.1, MW441215.1, MW441216.1, MW464122.1, MW464123.1, MW440988.1, MW440989.1, MW440990.1, MW440991.1), Germany (EU004820.1, AY702695.1), Turkey (PP823908.1), and Poland (AY603536.1) and of *P. moraveci* from the Czech Republic. Based on the evidence presented in this study and our new understanding of *P. moraveci* in red kites in the UK, it is considered likely that at least some red kites translocated from populations in England and Wales to Spain will be infected with *P. moraveci*. Molecular characterization of *Porrocaecum* isolates from Spanish red kites is required to determine the genuine occurrence of these two parasite species in Spain and accurately assess whether *P. moraveci* could pose a novel disease risk to destination populations of red kites and other raptors in Spain as a consequence of translocation. As such, reexamination of the *P. angusticolle* samples from these studies may be warranted to confirm identification of these parasites.

A small number of assumptions were necessary during the retrieval and examination of archived adult helminths from the case birds in this study. The red kites sampled were found dead and submitted to the DRAHS project for PME, representing a potential source of bias when working with endangered species and wild populations. Such convenient sampling is a common necessity when working with wild populations to assess intestinal pathogen loads. While it was not possible to assign a cause of death to many birds, the data generated do illustrate the range of parasites currently present within the red kite population of England and Wales. Additionally, it was assumed that all the adult GI helminths present had been detected by the attending wildlife veterinary pathologist during the PME of each red kite. As several of the red kites were noted in the PME reports as having been in an advanced stage of decomposition when they were submitted, it is likely that some sections of the GI tract and associated parasites were autolyzed before the PME took place and not detected or extracted for archiving. Additionally, it should be noted that the method of parasite detection utilized in this study is fairly non-specific and insensitive. As such, it is possible that parasites that burrow into the tissue and near-microscopic helminths were missed during the gross examination of the gastrointestinal tract.

Similarly, the relatively low success rate for PCR amplification may have been influenced by degradation of the genomic DNA used as the template prior to extraction. Further, we noted during microscopic examination of the archived adult nematodes that, for some specimens, only a subsection of the full body of the parasite was present. For the purpose of this study, it was assumed that parasite subsections represented separate individual nematodes, which may have influenced accurate conclusions regarding the parasite burden within each case bird. This limited the number of nematodes for which it was possible to obtain full morphometric data. Finally, and most importantly, it was assumed that all parasite species found had been previously described in the literature, as highlighted by the recent description of *P. moraveci* (Gu et al. 2023). While all samples identified to species level in this study have been annotated as *P. moraveci*, it is possible that other *Porrocaecum* species were either present and not detected, or present in the red kite population and not sampled. As morphological identification was limited to the genus level, final species identification relied on the existing genetic sequences in GenBank.

Red kites are considered migratory birds, but the degree of migration varies by individual, with some establishing year-round home ranges (van der Wal et al. 2015). There have been no studies evaluating whether individuals from the UK population cross the English Channel and interact with the larger European population; some studies are showing that fewer individuals are migrating in European populations (Garcia-Macia et al. 2021, 2022; Mattsson et al. 2022; Panter et al. 2022). While migration between the Great British population and the greater European populations has not been established, it is not currently possible to rule out some crossover between these individuals and their subsequent parasites.

This study focused on the identification of the GI helminths in free-living red kites but did not assess the association between these infections and pathological changes. Further studies would be required to understand whether infection with *P. moraveci* may be associated with disease and examine population effects in red kites. Continued health surveillance and identification of parasites detected in both source and destination red kite populations is recommended in order to iteratively inform disease risk analysis for conservation translocations of the species between England and Spain.

## Data Availability

All genetic sequences isolated in this study are available from GenBank under the accession numbers OR359357-OR359369 (ITS-2) OR356064-OR356077 (COI) and PQ634575-PQ634581 (28S). Data relating to post-mortem examination reports and images of parasites analyzed in this study are available upon request. Remaining parasites not utilized for PCR are archived at ZSL’s main campus in London.
